# A Challenging Yet Motivating Journey: The Experiences of Young Adult Parents With Serious Mental Health Conditions in the USA

**DOI:** 10.3389/fpsyt.2022.814185

**Published:** 2022-03-14

**Authors:** Kathryn Sabella, Amanda Baczko, Ian A. Lane, Laura Golden, Emma Pici-D'Ottavio, Murron O'Neill

**Affiliations:** ^1^Transitions to Adulthood Center for Research, Implementation Science and Practice Advances Research Center (iSPARC), Department of Psychiatry, University of Massachusetts Chan Medical School, Worcester, MA, United States; ^2^Sociology Department, University of Massachusetts Boston, Boston, MA, United States; ^3^School of Social Work, Boston University, Boston, MA, United States

**Keywords:** emerging adult, mental health conditions (MHCs), young adulthood (18 years older), parenting with mental illness, qualitative

## Abstract

In recent decades the average age of becoming a parent has increased, the rate of teen pregnancies has decreased, and a new developmental period of emerging adulthood is marked by diverse pathways into adulthood. Today, those who become parents in young adulthood (18–24 years old) and their children may be vulnerable to poor outcomes observed in teen parents (13–19 years old) of previous generations. Young adults with serious mental health conditions (SMHC) who encounter additional challenges navigating young adulthood and tend to parent earlier than their peers may be at particularly increased risk of poor outcomes. To date, little research has been done to understand the experiences of young adult parents, especially those with SMHC. This study describes themes from qualitative interviews with 18 young adults with SMHC in the United States who became parents before the age of 25. Life story narrative interviews, conducted mostly by young adults with lived experience, asked participants to describe their parenting and mental health experiences and their school, training, and work experiences. Participants described the challenges of simultaneously parenting young children and managing a mental health condition, experiences of discrimination, and fear of future discrimination related to their mental health condition. However, parents also expressed that their children motivated them to maintain recovery and build a good life for their family. This is the first study to qualitatively explore the experiences of young adult parents with SMHC. While many of these findings align with prior qualitative research on mothers with mental illness, by exclusively focusing on individuals who become parents earlier than their peers and including father experiences, this research adds to our understanding of how individuals simultaneously navigate parenting and managing a serious mental health condition. These findings should inform larger-scale research studies on the experiences and outcomes of young adults with SMHC who become parents in their late teens or early twenties. A better understanding of their experiences should inform public mental health services that incorporate parenting as an important element of an individual's personal recovery model.

## Introduction

As the average age of becoming a parent has increased in recent decades, those who become parents in young adulthood (18–25 years old) and their children may be vulnerable to poor outcomes like those of teen parents (13–19 years old) and their children from previous generations. Particularly at risk are young adults with serious mental health conditions (SMHC)^1^ who often have poorer education, employment, and housing outcomes and tend to become parents earlier than their peers. By virtue of their age and their mental health condition, young adults with SMHC, and their children, may be at higher risk for negative outcomes (1, 2). Thus, targeted services to support young adult parents with SMHC are imperative. However, such services will only be effective if they are informed by the lived experiences of young adult parents with SMHC. To date, research on this population is scant. This paper will describe results from qualitative interviews with young adults with SMHC who became parents in their teens or early twenties. This information should be used to inform public health and mental health programming.

### Teen Parents and Their Children: Historical Health Outcomes and Public Response

In the 1990s, the birth rate for females ages 15–19 peaked at 61.6 births per 1,000 women (3). During this time, research increasingly showed that teen parents and their children were at increased risk of several poor outcomes. Teen parenting was linked to increased rates of poverty and decreased social well-being in parents (4, 5). Additionally, the educational pursuits of teen parents were often negatively impacted. In 2008, over 90% of the general population received their high school diploma by age 22 compared to only 50% of teen mothers (4). Completing continued education was even less likely; only 10% of teen mothers pursued and completed an associate or bachelor degree program (4). Children born to teen parents experienced disproportionate rates of health and development issues including low birth weight and infant mortality (6, 7). Studies also showed that children born to teen parents were more likely to be victims of neglect with rates of maltreatment estimated at over 50% (8, 9). As children of teen parents grew up, they also experienced higher rates of teen pregnancy, poorer educational outcomes, and were at increased risk of substance use disorders and SMHC (10, 11).

In response, several public health initiatives emerged to reduce the rate of unplanned teen pregnancies (12). For example, the National Campaign to Prevent Teen and Unplanned Pregnancy, launched in 1996, developed and distributed public health knowledge to inform the conversation on teen pregnancy and parenting (13). The Teen Pregnancy Prevention Program (TPPP), an evidence-based program enacted in 2010, distributes grant funding to programs across the US aimed at preventing teen pregnancy (14). TPPP programs and many other successful public health initiatives have helped to develop and disseminate comprehensive sexual education and provide funding for family planning and young adult development (14). Many of these initiatives boast success in reducing teen pregnancies; teen pregnancy rates have steadily declined and currently hover around 7–16% (15).

### Young Adulthood and Young Parenthood in the 21st Century

Over the last two decades, the period of young adulthood (broadly ages 18–30) has evolved into a newly understood life-phase known as “emerging adulthood” (16). Normative activities that previously marked one's transition into adulthood (e.g., completing education, securing stable employment, marrying, and becoming a parent) now typically occur several years later than in previous generations (16). In the United States, a 2015 report by the National Academy of Medicine (formerly the Institute of Medicine) and the National Research Council concluded that young adulthood (17–25) is a critical developmental period marked by increasingly diverse pathways (26).

In the United States, the lengthening transition from adolescence to adulthood is evident in patterns of employment, education, and living status. Between 1960 and 2018 the percentage of recent high school graduates who went on to higher education increased from 45.1 to 69.1 percent (17). In recent years, even before the COVID pandemic, the rate of young adults living at home with their parents was the highest rate since the Great Depression (18). Emerging adulthood has also been marked by a steady increase in the average age of first birth. Between 1970 and 2000, the mean age for women having their first live birth in the United States increased by 3.6 years (19). That rate has accelerated over the last two decades; census data show that between 1994 and 2018, the average age for women having their first live birth increased another three years from age 23 to 26 (20). This trend is evident across other datasets (21). The average age men are becoming fathers has also increased by several years (22, 23).

At the end of the 20th century, poor outcomes among teen parents and their children, such as those described earlier, reflected concerns that teen parents did not have sufficient physical and emotional maturity to provide effective care for a child and that they may not have adequate economic resources (24). In the U.S., public health officials framed these data to portray teen pregnancy as a social problem that needed to be addressed. However, as the average age of first birth has increased over this time, and a unique developmental period known as emerging adulthood has evolved, individuals who become parents significantly earlier than their peers, even if not technically “teen parents,” may face challenges similar to those of teen parents in earlier generations.

Historically, research on teenage parenting utilized an absolute age cut-off (i.e., the age of 20) to define a group of parents most at risk for poor outcomes. However, the timing of what constitutes “early” parenting differs by society and over time. A recent cross-national comparative study looking at educational differences in early childbearing defined “early childbearing” as the age by which 20% of first births have occurred to women in a given birth cohort and country (24). In this definition, “early childbearing” is defined in relative terms, i.e., in relation to the normative timing of childbearing within a given society or cohort. The authors argue that since teenage childbearing is relatively rare (and has been declining) in many countries, its utility as an absolute measure of “early childbearing” is waning. Taken together, elongated and diverse pathways from adolescence to adulthood and the increasing average age of first birth necessitate an updated definition of what constitutes a “young parent” or “early childbearing” in the United States. Following this logic, the high-risk category that was previously defined as “teen parents” should now be redefined in more relative terms. For instance, in the United States, only 30% of pregnancies happen prior to the age of 24 (25). Accordingly, the outcomes and needs of young parents in their late teens *or* early twenties might be poorer relative to people who become parents later in life.

### Young Adult Parents With SMHC

Pathways through the transition to adulthood are more diverse and increasingly difficult to predict (26). Most mental health conditions are diagnosed by age 24 (27) and it is estimated that between 5 and 7 million young adults (ages 16–25) in the United States have serious mental health conditions (SMHC) (28, 29). The term “serious mental health conditions” (SMHC) is used to be inclusive of mental health diagnoses of “serious emotional disturbance” in childhood and diagnoses of “serious mental illness” in adulthood. SMHC includes schizophrenia-spectrum disorders, bipolar disorder, and severe forms of depression, anxiety, and post-traumatic stress disorders. In the U.S., mental health conditions are the cause of 45% of the burden of disability in older youth and young adults (26). Compared to their peers, young adults with SMHC experience lower rates of high school graduation (30), higher rates of college drop-out (31, 32), and are less likely to be employed (31, 33–35). Youth and young adults with SMHC are over-represented in homeless populations (36, 37) and have high rates of co-occurring substance use (28).

On average, young adults with SMHC become first-time parents at an earlier age than their peers without SMHCs (2, 38, 39). The average age of becoming a parent is currently 26 years old for females (40) and 31 years old for males (22). Meanwhile, depending on the diagnosis, the average age of becoming a parent for women living with SMHC ranges from 19 to 22 years and from 24 to 25 years of age for men with SMHC (2). Prior research shows that among young adults ages 18 to 26 years, 29% of those with SMHC are parents of at least one child, compared to just 19% of their peers without SMHC (39). Based on estimated mental illness prevalence rates cited earlier, we estimate there are 1–2 million young adults with SMHC who are parents of young children in the United States.

Based on the limited research that is available, parents with serious mental health conditions (SMHC) of any age face a myriad of challenges as they navigate adulthood. Parents with SMHC experience higher rates of poverty, lower employment rates, and higher rates of enrollment in Supplemental Security Income (SSI) or Social Security Disability Insurance (SSDI) than parents without SMHC (1, 2). They are more likely to be living without partners and managing parenting and financial responsibilities without spousal support (2). Parents with SMHC face the additional barrier of stigma and discrimination when disclosing their mental health diagnosis (41). As a result, parents with SMHC may not be forthcoming about their challenges or needs (42).

Research has found that parents with SMHC often experience less positive relationships and poor attachment with their children compared to parents without mental illness (43–46). Although experiences can vary by many contextual circumstances (e.g., race/ethnicity, social class, severity of mental health needs), parental mental illness can also be associated with low family cohesion (47) and higher family conflict, which can potentially lead to overall more problematic behaviors in the children (48). For instance, children of parents with SMHC can experience increased levels of distress, deviance, and heavy drinking in adolescence (49) and demonstrate less academic competence (50). Children of parents with mental illness may experience negative emotions such as anger, fear, and sadness more frequently, which may lead to additional internalizing problems (51, 52) or externalizing problems (53). One study found that about one-third of children of parents with SMHC are at risk of developing a serious mental illness themselves (54).

### The Need to Better Support Young Adult Parents With SMHC

Taken together, young adult parents with SMHC may be particularly at risk for poor outcomes by virtue of both their age (relative to average age of becoming a parent) and their SMHC. Young adult parents are often unable to engage in age-normative activities (e.g., employment and post-secondary education), which are primary pillars of their mental health recovery and long-term career trajectories (55). Parenting in young adulthood creates additional psychological strain for those with preexisting SMHC. The existing research, mostly with mothers with mental illness, illustrate how the normal stresses of parenting are exacerbated when also struggling to manage one's own mental illness (56, 57) and managing competing identities of “mother” and “person with mental illness” (58). Furthermore, mothers with SMHC struggle to juggle the practical demands of parenting and stigma while taking care of themselves and their condition; maintaining consistent engagement with mental health services appears to be particularly challenging (56–58). Regardless of the challenges, many mothers with mental illness have reported that parenting and caretaking have positive effects on their overall well-being and that they feel a lot of pride for being a parent (42, 59, 60). However, most of this exploratory research was limited to only the experiences of mothers, and also mothers with mental illness who were in their late 20s or 30s.

Outcomes of children of parents with SMHC are the result of a complex web of risk and protective factors in the family and social environment (61, 62) that can create a particularly complex mental and public health issue with no easy solution (63). Existing interventions have been found to significantly reduce the risk of children exhibiting internalizing symptoms or developing the same mental illness as their parent (64). However, many promising interventions are still being tested, are not widely disseminated, and have not been implemented in diverse settings (65).

To be effective, interventions to support parents with mental illness need to be informed by their needs and experiences. While there is some qualitative literature on parents with mental illness, especially mothers, descriptive and exploratory research on the experiences of young adult parents with SMHC is almost non-existent. The current study aims to describe the experiences of young adults with SMHC who became parents at age 25 or younger (i.e., young parents) and how they navigate young adulthood while managing a SMHC and parenting young children. Findings from this study will invaluably inform efforts in the public health sector to adequately support and tailor services to address the unique needs of this population.

## Materials and Methods

### Community Based Participatory Research

This study applied principles of Community Based Participatory Research (CBPR) by collaboratively and equitably involving community members and researchers in all aspects of the research process (66, 67). The center that housed this research employs young adults with lived experience of a mental health condition as research coordinators. This research was motivated by the lived experience of a young adult staff member who was parenting a young child. She and other young adult staff contributed to the iterative design of the study, led recruitment activities, conducted most of the interviews, and participated in the analyses, interpretation, and dissemination of study findings, including the writing of this article. We also acquired input from the Youth and Young Adult Advisory Board (YAB) affiliated with the research center that housed this study. The board is comprised of 8–10 young adults with lived experience of a mental health condition from across the U.S. They met monthly to provide input and feedback to all center projects. The principal investigator and research coordinators met with the board several times to obtain input on the goals of the study, get feedback on recruitment activities and materials, and review, pilot, and edit the interview script.

### Participants

The young adult parents in this paper represent a subgroup of participants from a larger qualitative interview study of 61 young adults with SMHC in the greater Boston and Central Massachusetts area. The purpose of the larger qualitative study was to explore and describe the experiences of navigating employment, education, and training in young adulthood while managing a mental health condition. In order to be eligible to participate in the larger study, participants had to be between the ages of 25–30, have some school or work experience, and report having been diagnosed with a serious mental health condition (e.g., major depression, anxiety disorder, bipolar disorder, schizophrenia) that was diagnosed by a medical provider at or before age twenty-two. Research and policy relative to individuals with mental health conditions often distinguish the level of impairment or seriousness associated with that condition (e.g., no impairment, moderate impairment, or serious impairment). Unfortunately, definitions of what entails “functional impairment” are not universal. The research team collectively identified several indicators that the young adult's ability to function was impaired at some point due to their mental health condition. As a result, participants had to endorse one of the following experiences: receiving intensive outpatient or inpatient mental health treatment, receiving services by the Massachusetts Department of Mental Health, receiving formal special education services because of the mental health condition (includes have an IEP or a 504 plan), or having to take a formal leave of absence from school or work due to their mental health condition. To explore our research aims regarding young parents with SMHC we (a) extended the age eligibility range down to age 22, and (b) oversampled young adults who had at least one child 12 months or older living with them for at least 50% of the time for a minimum of six consecutive months. Exclusion criteria for the study included being unable or unwilling to provide informed consent or being unable to read and understand written and spoken English.

### Recruitment and Procedures

Participants were recruited via flyers and electronic announcements shared with community mental health agencies, clubhouses, and Department of Mental Health providers. The center shared the research opportunity on social media and through electronic newsletters. It is important to note that these more general recruitment strategies resulted in a sizable sample of young adults but did not reach a sufficient number of young adult parents with SMHC. After consulting with our advisory boards and providers in the area, we broadened our recruitment to more general social service agencies (e.g., those served by agencies other than Department of Mental Health) and homeless shelters. These additional recruitment strategies led to the recruitment of more young parents with SMHC. Study flyers offered contact information to provide more information about one's involvement and voluntary participation in the study. Interested young adults were contacted by research staff and completed brief phone screenings to assess eligibility. If deemed eligible, research staff scheduled their interview. All procedures were approved by the Institutional Review Board at the University of Massachusetts Chan Medical School.

### Data Collection

The research team conducted semi-structured qualitative interviews in 2016 with study participants who identified as young adult parents (*n* = 18). Most interviews (*n* = 17) were facilitated by young adult research staff members with lived experience of a mental health condition and took about 90 min to complete. The remainder were conducted by the Principal Investigator. Interviews took place in-person in community settings, at a convenient location identified by the study participant. Interviewers reviewed informed consent procedures, asked participants to complete a brief demographic survey, and conducted the semi-structured interview. Two participants declined being recorded and instead detailed qualitative notes were taken by the interviewer. Participants were each compensated with a $30 gift card upon completion of the interviews.

The interview script was modeled as a life story narrative interview (68). The interviewer asked the participants to describe, (a) their educational, vocational training, and employment experiences, (b) how these activities occurred over time, and (c) how contextual life circumstances and experiences (e.g., family history, experiences with SMHC, major life events) influenced those activities. Participants who were also parents were also asked questions about their children, their parenting experiences, how their lives changed after becoming a parent, custody and living arrangements, and challenges or supports they experience related to parenting. Interviewers were instructed to ask about a certain set of mental health related experiences if details were not offered (e.g., suicidality, inpatient hospitalizations).

### Data Analysis

Audio-files or interview notes were transcribed and entered into Dedoose Qualitative Coding Software (69). A team of three coders inductively created a qualitative codebook and coded all interviews using exploratory and grounded theory approaches (70). In the initial wave of coding, the coders read through a third of the transcripts to identify and discuss major topics and themes that would form the basis of the codebook. Each member of the research team then descriptively coded some of the same transcripts independently and met for regularly scheduled sessions to compare codes and achieve consensus on the further specification of codes. The codebook was continually refined by repeating this process until inter-rater reliability was consistently attained (*k* = 0.80). The remaining transcripts were coded by the first and second author. In the final wave of coding, the first and second authors completed thematic coding and extracted and chronologically described information about each young adult parent's parenting experiences (e.g., custody status, living arrangement) and accompanying employment and education activities (e.g., start date, end date, description of the experience, why ended).

## Results

### Participants

Of the 18 young adult participants, 83% identified as female and the majority were white, non-Hispanic (55.6%) (Table 1). The mean participant age at the time of the interview was 26 years old. The majority of participants were never married (55.6%) and half the sample (50%) were living independently without the live-in support of their own parents or other extended family members. Two participants were living in homeless shelters with their children and one participant was living in a group home after having been released from prison the month before. For almost half of the participants (44.4%), a high school diploma or GED was the highest level of education they had ever obtained. About a third of participants (38.9%) had attempted college but not been able to complete a degree (38.9%). Only three participants completed a post-secondary degree. Most participants (55.6%) reported making < $10,000 per year (not including benefits).

**Table 1:** Demographics.

**Variable**	**N**	**%**
**Gender**		
Female	15	83.3
Male	3	16.7
**Race/ethnicity**		
Non-Hispanic white	10	55.6
Non-Hispanic black	2	11.1
Hispanic (Puerto Rican, Columbian)	3	16.7
Mixed or other race	3	16.7
**Age at time of interview**		
22–23	4	22.2
24–25	3	16.7
26–27	6	33.3
28–30	5	27.8
**Marital status**		
Never married	10	55.6
Currently married or cohabitating	5	27.8
Divorced or separated	3	16.7
**Living arrangement**		
Living independently	9	50.0
Living with their own parents or extended family	6	33.3
Living in homeless shelter or group home	3	16.7
**Educational attainment (at time of interview)**
High school graduate or GED	8	44.4
Some college, degree not obtained	7	38.9
Associates degree	2	11.1
Master's degree	1	5.6
**Annual personal income in last year (not including benefits)**
$0–$10,000	10	55.6
$10,001–$20,000	5	27.8
$20,001–$30,000	2	11.1
$30,001–$40,000	0	0.0
$40,001–$50,000	1	5.6

All participants except one reported receiving multiple mental health diagnoses. The most frequently self-reported mental health conditions were anxiety (83.3%) and major depression (72.2%), followed by PTSD (50.0%) and bipolar disorder (50.0%). Approximately 30% of participants had engaged in a suicide attempt or self-harm. Twelve participants had been hospitalized overnight due to their SMHC and of those 12, half had been hospitalized five or more times (Table 2).

**Table 2:** Mental health characteristics.

**Variable**	**N**	**%**
**Serious mental health conditions**		
Anxiety	15	83.3%
Major depression	13	72.2%
Post-traumatic stress syndrome (PTSD)	9	50.0%
Bipolar disorder	9	50.0%
Schizophrenia	1	5.6%
Schizoaffective	1	5.6%
Eating disorder	5	27.8%
Borderline personality disorder	1	5.6%
**Other co-occurring conditions**		
Attention deficit hyperactivity disorder	3	16.7
Substance use disorder	1	5.6
**Ever engaged in suicide attempt or self-harm**		
Yes	6	33.3
No	12	66.7
**Number of overnight psychiatric hospitalizations**
None	6	33.3
1–2	3	16.7
3–4	3	16.7
5–10	4	22.2
10+	2	11.1

Most participants identified as mothers but three fathers were included in the sample (Table 3). Some participants gave birth to their first child prior to the age of 20, thereby (40%) fitting the traditional definition of “teen parents” who had their first child before age 20. Two parents had more than two children (11.2%) and overall, children's ages (not shown) ranged from several weeks to 12 years old. The majority of participants had their children living with them the majority of the time. None of the fathers lived with their children the majority of the time, and only one of those had visitation rights.

**Table 3:** Parenting characteristics.

**Variable**	**N**	**%**
**Parental role**
Mother	15	83.3%
Father	3	16.7%
**Age at which participant first became a parent**
Under 20 years old	7	38.9
20–24 years old	11	61.1
**Number of Children**
1	9	50.0
2	7	38.9
3	1	5.6
4	1	5.6
**Proportion of time spent living with their children**
Most of the time (>75% of the time)	15	83.3
None of the time, visitation only	1	5.6
None of the time, no visitation	2	11.1

### Qualitative Findings

Several themes emerged from the qualitative interviews specific to parenting including the strategies that young adult parents employ to manage their mental health, how children act as a motivator for recovery, and experiences of stigma and discrimination.

#### Managing Symptomatology of MHC While Parenting

Participants often reported that mental health symptoms impeded their ability to fully concentrate on their children and be present. One participant described how their symptoms of depression would often get in the way of their ability to engage in play with their child: “I start slacking on my kids. And like I know like he'll get restless with me, and he'll be like “mommy, you're not playing with me anymore. You're not doing anything with me.” And that kind of like puts it into perspective.” In this way, children also helped increase their parent's cognizance of their mental health symptoms and serve as a reminder of when and how mental health symptoms got in the way of engagement.

Some participants described how the stress of parenting can trigger an increase in their overall mental health symptoms. For one participant, parenting a child at the age in which they themselves had experienced a traumatic event triggered an increase in PTSD symptoms: “My PTSD would kick in because I would think about when I was his age, like what my parents were doing to me at that time.” Another mentioned, “I feel like when he is sad or when he is sick, my anxiety is like triggered by that. It's like I try to do as much as I can to be able to help him or the situation because I feel like… I didn't have that.” To cope with increased symptoms and feelings of stress, some participants would be tempted to look to negative coping skills, like drug use, to manage:

But with him, like I get aggravated also a lot……being with him in maybe such a close range, right there in the same room, like it gets overwhelming……before when he would aggravate me, I would feel like my triggers, and I would want to use. I'd be like I just want to get high. He's just so annoying. I don't want to deal with this.

Mental health symptoms would be triggered by the stressors of parenting, and in those moments, negative coping skills were harder to avoid.

When participants tried to take care of their mental health and practice self-care, feelings of guilt often arose. Taking time out to receive treatment or to work on symptom management was difficult:

Yeah, it's hard… being a parent. And having kids and having to take care of them. And then having to stop everything because I needed to be hospitalized. It's very… I've always kind of had a little bit of guilt. I feel like in the back of my mind, every time I get hospitalized because it's like my—wherever what I'm supposed to be doing is just going to have to stop until I come back. And that can be stressful.

Others reported that their mental health got in the way of putting their child first, and touched on the associated feelings of guilt that came with it:

For me, it's sad, but it makes it harder for me to put my child first. And makes it harder for me to focus more on his well-being than what I want to do for myself. Which sounds really bad. It sounds really sad. But it's like if you want the honest to God truth, that's the honest to God truth. And I wish that it would be better. And I wish that I could put more focus on to him in making sure that he's good before anything else. But sometimes it's just like my brain won't let me do it. It's just like I come first. Which I don't want to. He's my baby.

This participant seemed to battle the overwhelming feeling that putting themselves first was selfish.

#### Children as Sources of Motivation and Recovery

While participants reported various obstacles to managing their mental health while parenting, the act of parenting itself was described by many as a primary motivator to initiate and maintain their mental health recovery. Participants reported that prior to becoming a parent, they were the ones most often influenced by their decisions in life. However, after having children, they needed to recognize that their behaviors and decisions, good or bad, would directly influence their children. This realization often acted as a motivator to power through the difficult times. Says one mother,

After I had my son, it's been like a situation where I refuse to like not [to] be able to provide for my son. And then being without me and it's not just me anymore, my family, you know? Even though I have my depression and my anxiety that was like a weight on my legs. I still go forward.

In some cases, participants described their children as a positive source of distraction, providing them the ability or need to focus on something other than the negative impact of their mental health:

And I have experienced dealing with the schizophrenia with them, and it's—most of the time they kind of are able to distract me. So, I can kind of focus on them and not worry so much about what's going on in my head.

Participants recognized the desire for a healthy distraction from their ongoing mental health symptoms, and parenting was sometimes cited as that source of refuge.

Other participants described their children as the motivator needed to embrace their recovery and persevere. Three participants shared experiences of suicidal ideation and how the need to be present for their children was strongly related to a sense of purpose, which helped provide a reprieve from acting on these thoughts. One participant in particular spoke about a shift in mindset taking place after almost being hospitalized for suicidal ideation:

You know I kind of like came home, put my foot down, and you know kind of did what I had to do. Because honestly, I don't want to leave my kids. You know because nobody will take care of them the way that I do.

Another participant described the need to look outside of themselves to not act on their ideation, and instead look to the impact on their family:

But when I get those thoughts, like I need to think about what is there to look forward to? And that's easier to think about when you're thinking outside of yourself, at least for me. My daughter made me, especially with her dad not being stable… I was always like… it would be selfish for me to leave because then where would that leave her.

One parent summed up their belief about the journey of parenting with a mental health condition, and offered advice and words of inspiration to other parents who should find themselves in a similar situation:

That having a mental illness and being a parent is possible. You know, and you don't have to be like ashamed. And I've met a lot of people who have told me—who have had like bad things happen to them. They're afraid that they won't be good parents. And I mean everything depends on the person, but I mean a person who is nervous about it, most likely they notice it. And I think that shows that they don't want that to happen. And I think just because you have like PTSD or ……something. It doesn't mean that your child is going to go through that with you. Like it doesn't mean you're bad. Like it doesn't—you know, like you can still be a great parent regardless of what your mental illness is.

Despite experiencing increased challenges and negative impact on mental health, participants still described the value of parenting on their recovery and were able to balance feelings of inadequacy with reminders that experiencing a mental illness did not equate to being a lesser parent.

#### Experiences of Discrimination and Feelings of Stigma

Some participants described discrimination and the resulting stigma they experienced as parents with mental health conditions. Often, these experiences were related to the ways in which others perceived their ability to be a parent. One participant described an experience of facing discrimination from a judge during an ongoing custody battle with their ex-partner:

I've definitely been discriminated against by the judge. It was horrible. They'd talk to me like I was five. They looked at me like I was a disease of a father. I wanted to be there for my kid but was never given the opportunity by the courts to do it.

This participant went on to describe how their mental health was used against them and threatened their parental rights:

His mother and I were in a relationship for not that long a time. We were both very, very, very young. She was 16 when she gave birth, turning 17. I was 19. And everything was great up until 8 months. We were brand new parents. Like everything's very hard, very stressful. And then the anxiety and the depression kicked in with wondering can I do this? Can we do this together? And we were very unhappy together. And then stuff that I confided in with her during our relationship, when we decided to end our relationship, she used my mental health against me in court. And it ended up not turning out very well.

Another participant described the added stressor of trying to manage going to school while parenting, and the stigma they faced as a student with a so-called “behavioral problem”:

And, definitely with teachers and school, they would look at you like you were a problem. Like a behavioral problem. If you were—like if I was like crying in class, they singled you out. They would single me out. And they'd go *Oh [Name] is crying again. What's going on with you? Is there something wrong? Did I offend you*?

Discrimination against a person due to their mental health condition is common. But for many parents in this sample, the fear or experience of discrimination was even more pronounced because of their role as a parent.

## Discussion

This paper contributes to the current understanding of the experiences of young adult parents with SMHC and the potential impact of mental health symptom management, stigma, and discrimination on navigating a successful path to adulthood. In this sample of 18 young adults with SMHC who became parents prior to the age of 25, many described struggling to manage the symptoms associated with their SMHC and its perceived effect on their ability to engage as parents. Furthermore, mental health symptoms were often exacerbated or triggered by the stressors of parenting. Symptoms may also impede the ability to fully engage in parenting, straining the relationship between parent and child. At the same time, an overwhelming majority of young adult participants described their children as their primary sources of motivation to stay on path of mental health recovery and to “better themselves.” Children were described by their parents as the primary factor for pushing through when things felt overwhelming, and elicited feelings of pride and determination.

Despite the many disclosed benefits of parenting on mental health, young adult parents also shared the negative impact of disclosing mental health to others. It was clear that many young parents with SMHC had experienced or were afraid of experiencing stigma and discrimination as a result of disclosing their mental health condition. Participants described instances of discrimination in public settings, including court rooms and educational institutions. Discrimination prevents treatment, and it impedes recovery (55, 71). Within various social services there is a prevailing blame mentality and risk discourse in which parents with mental illness are constantly monitored and often easily suspected of abuse when their symptoms are acute (72). Parents that are forced into silence will not engage in needed services if accessing that care could lead to discrimination and potential loss of custody.

Taken together, most of our findings echo previous qualitative research with parents, mostly mothers, with serious mental illness (42, 56–58, 60). However, given the research in this area is limited and the changing cultural norms of emerging or young adulthood, these findings can still uniquely inform public health and mental health services and approaches for parents with mental illness. Most people with mental illness will not only become parents but become parents at an earlier age compared to their peers (2). The earlier services are able to intervene to support parents with mental illness, the more likely they will succeed in supporting them and their families over time. Furthermore, given young adult parents in this study endorsed parenthood as meaningful and beneficial to their recovery, it is important for services to approach parent needs from a place of encouragement and motivation rather than too heavily focusing on risks or needs. The United Nations recently identified youth, defined as the period from 15 to 24 years of age, as a period of vulnerability worldwide (73). Young parents (i.e., those who have children prior to their mid-twenties) are still susceptible to experiencing the economic, social and health disadvantages that were often reserved for teen parents in previous generations. Young adults who are managing serious mental health conditions may be further susceptible to poor outcomes.

### Implications

Findings from this study can help to inform public and mental health initiatives that more adequately meet the needs of young adult parents who are simultaneously managing their own mental health condition. First, participants in this study described feelings of guilt when having to put their parenting “on hold” to seek out needed mental health care. This is of no surprise, as a majority of young adult parents are single parents, without a co-parent to balance the weight of added responsibilities that parenting carries. Allocating transportation and daycare funds for young adult parents with SMHC could provide a platform for increasing access to care, as well as alleviating the feeling of burden placed on young adult parents to “do it all” without adequate support.

Young adult Access Centers or Drop-In Centers are an increasingly popular model in the United States aimed at supporting the mental health and overall career development of young adults with SMHC. Access Centers provide a non-judgmental safe space where young people can meet with peers and community-based mental health professionals to access mental health care, get basic needs met (e.g., laundry, showering, food) and focus on career development (e.g., resume building, GED practice) (74). Building upon the successful Young-adult Access Centers model, additional programming within Access Centers should be tailored to young adult parents with SMHC. Expanding programming to meet the needs of young parents would offer a safe environment where parents could bring their children and engage in peer support and parenting education and develop concrete tools to continue to foster a wellness recovery plan that emphasizes the parent-child relationship. Access Centers are designed to have flexible/evening hours and are structured in a way that lends well to providing childcare services. This is ideal for young parents who are often balancing parenting, employment, and education simultaneously and do not have the luxury of accessing needed care during traditional office hours. The Access Center approach has already begun to draw interest from young adult parents with SMHC; a recent analysis showed that within a Massachusetts Young Adult Access Center, young parents represented 10–15% of all young adults served (74).

Services aimed at supporting young adult parents with SMHC should attempt to strengthen the relationship between parent and child and be cognizant of the positive role that parenting can have in the recovery process. Many public health initiatives aimed at teen parenting in earlier generations focused primarily on the negative outcomes and risks associated with teen parenting. Similarly, parenting with a mental health condition is often seen as an impediment or liability to both the parent and the child. However, it is clear from this data that parenting may foster resilience in young adults with SMHC and can be capitalized on as a positive source of motivation and a tool to maintain recovery. Peer support services, tailored for young adult parents with SMHC, may be part of the solution. Peers offer special knowledge, drawn from personal experience, as a unique resource to help navigate the very practical day-to-day challenges that parents encounter when raising children and navigating treatment and recovery (75). Providers, policy makers, and practitioners need to recognize how young adult parents with SMHC are motivated by their children and capitalize on that motivation.

While many mental health services prioritize the medical model of recovery (i.e., the reduction or management of symptoms), a broader more personalized recovery model would be more supportive of parents with SMHC, especially those younger and earlier in their mental health recovery journey. Personal recovery has been defined as recovery that aims to emphasize the ability to lead a meaningful, purposeful life, with or without ongoing episodes of mental illness (76). One brief family support intervention in Australia, Let's Talk About Children, aims to enhance the recovery journey of parents with mental illness by acknowledging and addressing the parenting life domain (77). Research on the effectiveness of this intervention is underway, but the model's approach to parenting as a “value-add” in one's personal recovery journey is a promising practice in light of our current findings. Nicholson et al. (78) have adapted Let's Talk into the ParentingWell Practice Profile to support mental health practitioners in implementing family-focused practice approach with adults with mental illness. An initial pilot of the ParentingWell Learning Collaborative in Massachusetts provided preliminary support for the feasibility and impact of the model (79). Future research and evaluation efforts should also explore necessary modifications of this recovery intervention to fit the unique needs of young adult parents with SMHC.

Additionally, stigma and fear of discrimination are real and can impede young adult parents' willingness and ability to seek adequate care for themselves or their children. An ongoing yet silent threat of removal of their children prevents parents from being forthcoming about their needs or stressors, which in turn negatively impacts their mental health, their parenting, and their children (42). The general public and professionals within social services are in need of training and education to dispel myths about living with a mental health condition and its impact on one's ability to parent. Research tells us that when people share their stories of recovery with the public, it encourages people to challenge their negative beliefs and assumptions about mental health (55). Providing increased education on mental health cultural competency to professionals in direct family support positions (e.g., court officials, departments of children and families) could alleviate discrimination in care settings where young adult parents may fear disclosure.

Finally, providing increased access to comprehensive and confidential parental mental health screenings in community-based parent service settings (e.g., parenting classes and support groups) and general healthcare settings could help to close the gap in reaching parents who are fearful of being stigmatized when approaching mental health services directly. Young adults with SMHC have some of the lowest mental health help-seeking rates compared to other age groups and often cite stigma and embarrassment as primary barriers to help-seeking (80). For young adult parents with SMHC, the additional fear of custody loss may exacerbate the desire to steer clear from formal services, and in turn, enhance the desire to seek support from more informal peer services. However, despite grappling with these systemic and environmental barriers to psychological care, many young adults access primary care on an annual basis (81). Increasing training and funding to general health care spaces already accessed by this population would contribute to increased public health services coverage, while supporting and honoring the comfort and safety of young adults with SMHC.

### Limitations

Limitations of this study include a lack of racial diversity in the sample. However, the sample's racial composition is not that different from that of Worcester County, MA where the majority of the sample resided (82). A lack of diversity limits generalizability as does a small sample size. The sample for this study was pulled from a larger study sample (*n* = 61). While we successfully oversampled to ensure about 1/3 of the full sample were young parents, given the diversity of pathways through young adulthood, these findings may not fully represent the full range of experiences of young adults with SMHC who are parents. The experiences of young adult parents and their children are highly contextualized and can vary on multiple characteristics including race, social class, and severity of mental health condition. However, these findings should inform a larger prospective study that will better understand the experiences of this population. Finally, only three of the participants were fathers. The experiences of fathers with mental illness have been largely missing from the literature and while this represents a small contribution, more research is needed to understand fathers with mental illness of all ages, especially those who are young and may not have full custody of their children.

## Conclusion

Given increased recognition of a period of “emerging adulthood,” young people who become parents in young adulthood (18–24 years old) and their children may be vulnerable to poor outcomes like those of teen parents (13–19 years old) from previous generations. Particularly at risk are young adults with serious mental health conditions (SMHC) who often have poorer education, employment, and housing outcomes and tend to parent earlier than their peers. Taken together, by virtue of their age and their mental health condition, young adults with SMHC, and their children, may be at higher risk for poor outcomes. This was the first qualitative study to explore the experiences of young adults with SMHC who live in the United States and became parents prior to the age of 25. These young adult parents described the challenges of simultaneously parenting young children and managing a mental health condition, experiences of discrimination, and fear of future discrimination related to their mental health condition. Like prior qualitative research with mothers with mental illness, these parents also regarded their children as motivators for their recovery and important elements of their overall personal recovery. However, by exclusively focusing on individuals who become parents earlier than their peers, and including father experiences, this research adds to our understanding of how individuals simultaneously navigate parenting and managing their own mental health. These findings are relevant to public mental health services. Mental health services should be offered in low-barrier settings with convenient hours for young parents. Programming should foster resilience in young parents and incorporate their role of parenting as an asset in their personal recovery journey. The continued threat of discrimination due to mental health stigma is particularly poignant for parents and will continue to negatively influence their help-seeking habits if left unaddressed.

## Data Availability Statement

The raw data supporting the conclusions of this article will be made available by the authors, without undue reservation.

## Ethics Statement

The studies involving human participants were reviewed and approved by University of Massachusetts Medical School Institutional Review Board. The patients/participants provided their written informed consent to participate in this study.

## Author Contributions

KS is the principal investigator and lead author. AB contributed substantially in the writing the manuscript. LG and EP-D'O participated in data collection, analysis and manuscript preparation. IL and MO'N assisted in the writing and editing of the manuscript. All authors contributed to the article and approved the submitted version.

## Funding

This study was funded through a center grant to the University of Massachusetts Chan Medical School from the National Institute on Disability, Independent Living, and Rehabilitation Research (NIDILRR). NIDILRR is a Center within the Administration for Community Living (ACL), United States Department of Health and Human Services (HHS) (ACL grant# 90RT5031, The Learning and Working Transitions RRTC: PI, Maryann Davis, PhD).

## Conflict of Interest

The authors declare that the research was conducted in the absence of any commercial or financial relationships that could be construed as a potential conflict of interest. The handling editor JN declared a shared affiliation, though no other collaboration, with one of the authors KS, EP-D'O, and IL at the time of the review.

## Publisher's Note

All claims expressed in this article are solely those of the authors and do not necessarily represent those of their affiliated organizations, or those of the publisher, the editors and the reviewers. Any product that may be evaluated in this article, or claim that may be made by its manufacturer, is not guaranteed or endorsed by the publisher.

## Footnotes

^1^SMHC refers to having been diagnosed either with a serious emotional disturbance (often used in children) or a serious mental illness (often used with adults) as defined in the Federal Register [5/20/1993, FR, 58(96). P. 29422]. SMHC includes schizophrenia-spectrum disorders, bipolar disorder, and severe forms of depression, anxiety, and posttraumatic stress.
